# Tool Life Performance of Injection Mould Tooling Fabricated by Selective Laser Melting for High-Volume Production

**DOI:** 10.3390/ma12233910

**Published:** 2019-11-26

**Authors:** Mennatallah F. El Kashouty, Allan E. W. Rennie, Mootaz Ghazy

**Affiliations:** 1 Al Fouad Co. for Automotive Spare Parts, Alexandria 23722, Egypt; 2 Engineering Department, Lancaster University, Bailrigg, Lancaster LA1 4YW, UK; a.rennie@lancaster.ac.uk; 3 Industrial and Management Engineering Department, College of Engineering and Technology, Arab Academy for Science, Technology and Maritime Transport, Abu Qir Campus, Alexandria 21913, Egypt; mootaz.ghazy@aast.edu

**Keywords:** Rapid Tooling, additive manufacturing, Selective Laser Melting, injection moulding, tool inserts, automotive industry

## Abstract

Rapid Tooling processes are developing and proving to be a reliable method to compete with subtractive techniques for tool making. This paper investigates large volume production of components produced from Selective Laser Melting (SLM) fabricated injection moulding tool inserts. To date, other researchers have focused primarily on investigating the use of additive manufacturing technology for injection moulding for low-volume component production rather than high volume production. In this study, SLM technology has been used to fabricate four Stainless Steel 316L tool inserts of a similar geometry for an after-market automotive spare part. The SLM tool inserts have been evaluated to analyse the maximum number of successful injections and quality of performance. Microstructure inspection and chemical composition analysis have been investigated. Performance tests were conducted for the four tool inserts before and after injection moulding in the context of hardness testing and dimensional accuracy. For the first reported time, 150,000 injected products were successfully produced from the four SLM tool inserts. Tool inserts performance was monitored under actual operating conditions considering high-level demands. In the scope of this research, SLM proved to be a dependable manufacturing technique for most part geometries and an effective alternative to subtractive manufacturing for high-volume injection moulding tools for the aftermarket automotive sector.

## 1. Introduction

Selective Laser Melting (SLM) is an additive manufacturing (AM) process that produces three-dimensional (3D) functional metallic parts [1,2] directly from CAD data by selectively melting metallic powder using a laser beam, forming near net-shaped layered components that typically require post processing for surface finish improvement [3,4]. AM processes facilitate fabrication of geometrically complex components and freeform designs, as opposed to the limitations associated with conventional subtractive machining [5,6]. Despite these positive aspects, AM techniques continue to exhibit disadvantages that must be addressed and surpassed [7,8].

Studies have discussed an approach to improving AM techniques to provide a better-quality surface finish on fabricated metallic parts [9,10]. Currently in this context, Ahn and Yakout et al. [11,12] stated that none of the commercially available AM technologies has the ability to produce net-shaped components that require no further post-processing. Furthermore, Guo et al. [13] mentioned that parts fabricated by AM processes may require post-processing due to low dimensional accuracy and poor surface quality. Conversely, Gokuldoss et al. [14] reviewed that SLM technology tends to produce accurate parts or that minimal tolerance is required. Advances in AM are progressing to improve surface finish, dimensional accuracy, and durability; advances in machining research are also in progress [15].

Although affordable alternatives are sought after to avoid the use of tooling, in most part reproduction, rapid manufacturing is an alternative that has been unable to overcome the use of tooling as indicated by Wohlers [16]. Tooling continues to be essential to many industries for higher-volume production quantities because of the benefits of speed and cost. Tool making is a complex procedure and demands the use of high-end technology and skilled labour; therefore, industries are seeking out the use of Computer Numerical Control (CNC) machines in order to produce components with high quality despite the longer machining time and cost of manufacturing the tools [17]. However, recent research has proven the success of incorporating AM in the toolmaking process for low-volume production [18].

Researchers have shown that Rapid Tooling (RT) is a technique with great potential that aims to significantly reduce the product development cycle [19,20], eventually yielding cost and time benefits [21]. Wohlers [16] indicated that AM should not be overlooked as a technology that can produce tools, with significant potential to produce tooling inserts. There are two approaches to rapid tool manufacturing: direct and indirect tooling. Ding et al. and Au et al. [22,23] stated that direct tooling does not necessitate the production of a pattern, as tool inserts are produced directly. The use of each depends on the potential characteristics required by the manufacturers and the size of the production volume [24]. Contrary, indirect tooling necessitates the use of a master pattern that can be produced using an additive manufacturing method such as Selective Laser Sintering (SLS) or Stereolithography (SLA).

Long-term consistent tools should be capable of producing several thousands of parts before eventually wearing out. Levy et al. and Kruth et al. [25,26] highlighted the importance of tooling applications particularly for injection moulding, while other techniques such as sheet metal forming and forging dies were considered for low volume production. Previous successful studies were reviewed by Rahmati & Dickens [27] for low volume production of injection mould tooling. Kashouty et al. [28] presented a comparative study to assess additive and subtractive manufacturing technologies through fabricating two identical tool inserts that produced 500 injected components. Other studies focused on producing 500 components and subjecting the tool to severe stress and thermal conditions while performing the necessary tests to obtain the required data. Moreover, during the injection process, theoretical and analytical investigation of the tools were carried out. Additional studies by Xhang et al. [29] specified the durability of carbon fibre reinforced photopolymer tool inserts up to 2500 injections before a deterioration of the tool inserts was noticeably observed. This ‘soft’ tooling process was suitable for production volumes that range from 1000 to 10,000 cycles of injection moulding.

Other research analysed and reviewed the use of RT for the production of tools and dies, whether direct or indirect for low-volume or high-volume production, without conducting a more in-depth evaluation of the number of parts produced [30,31]. Ponche et al. [32] proposed a numerical chain based on a new design for AM methodology detailing both design requirements and manufacturing specificities, whilst Nagahanumaiah & Mukherjee [33] presented a systematic approach for manufacturability analysis of moulds produced by RT methods, the approach being founded on three phases: mould feature manufacturability; secondary elements compatibility; and cost effectiveness. The presented methodology not only assisted in RT process selection, but also facilitated the process of recognising minor adjustments to a tool design that eventually improves its manufacturability and cost. Ahn [11] presented research that investigated methods to overcome limitations of conventional tools in the context of energy consumption, environmental impact and material usage to develop eco-friendly tools. Machining time and cost is significantly reduced when compared to subtractive manufacturing approaches based on CNC machining for tool manufacturing [29]. Brooke [34] referred to Hopkinson’s argument that High Speed Sintering (HSS) will eventually displace CNC technologies for the production of components in high volumes. Achillas et al. [18] debated that AM technologies are not capable of replacing injection moulding for medium and high production volumes. However, RT could be incorporated for low volume production to achieve shorter lead-times and reduced production costs. Mahshid et al. [35] specified that advances in laser-based AM processes permitted fabrication of complex metal components that are impossible to achieve using subtractive processes alone.

Akula and Karunakaran [36] proposed that certain characteristics must be maintained for RT processes to ensure the success of manufacturing accurate tools. To ensure viability of AM technology, geometric and dimensional quality should be improved for rapid tools, whilst eliminating human intervention and reducing cost and time, to be as close as that attained in the case of conventionally manufactured tools [33]. Gu et al. [37] discussed the necessity of producing parts that meet the mechanical properties required by industry, hence, emphasising that the role of AM is towards functional components that serve industrial sectors. Flynn et al. [7] reviewed the most common approaches to finishing AM fabricated metal components through subtractive machining, thermal, chemical and electrochemical processing. Maamoun et al. [38] studied the effect of thermal post processing on the performance of SLM parts. Machining is generally used to improve dimensional accuracy in near-net shaping processes such as moulding. Additional context is reported within the literature for surface quality expectations of AM metallic parts. Spierings et al. [39] recommended finishing of AM components using CNC turning for selected types of steels to achieve the desired surface roughness. Löber et al. [10] used grinding, whilst Rossi et al. [40] were able to report the distinct variation in values of surface roughness between vertical and horizontal surfaces, that clearly signify the importance of build orientation. Zhang et al. [41] presented a study that focused on fabricating micro-structured injection mould tools for the production of thermoplastic microfluidic chips, however, signifying that surface finish and precision needs improvement.

Current research indicates that improving injection moulding cycle time is an important aspect when considering high-performance tools rather than the time taken to produce the tool [16]. Mahshid et al. [35,42] reviewed the possibility of achieving an alternative to manufacturing tools that is capable of producing a lightweight structure that potentially decreases material and manufacturing cost, and eventually leads to a decrease in production cycle time and increasing tool longevity. Interest over recent years is directed towards high-performance tools, however, only examples of low-volume production are given in recent literature. Therefore, more research must be oriented towards tooling for high-volume production and presenting the necessary means for investigating the outcomes. The research presented here focuses on the production of SLM tool inserts and assessing their durability and quality through high volume production of injection moulded components.

This paper considers the processes employed for fabricating four sets of injection moulding tool inserts, with a detailed description of the experimental work undertaken. After the experiments were conducted, the tool inserts were tested for durability and how they were used for the injection moulding of multiple thousands of products from each of the four tool inserts. The injection moulding process was performed in four stages. The four sets of tool inserts each achieved 10,000 injections whereupon the first tool insert was then removed. The remaining three sets of tool inserts reached 20,000 injections and then the second tool insert was detached. The same process was repeated for the remaining two sets of tool inserts and 30,000 injections were completed, after which the third tool insert was removed from the bolster. The last tool insert achieved 150,000 cumulative injections. Experiments were conducted prior to the injection process to inspect microstructure using a Scanning Electron Microscope (SEM), analyzing intermetallic carbide formation with a linked Energy Dispersive Spectroscopy (EDS) system, and hardness tests using Micro-vickers hardness tester to examine the influence and impact of the injection moulding process on the hardness of the material. Further experiments were required to be carried out after the injection moulding process was completed to ensure tool longevity in the context of hardness testing and measuring dimensional accuracy. Mechanical performance of an injection moulding tool insert such as tool hardness, wear resistance, surface roughness and dimensional accuracy significantly affects the production process. Therefore, this study investigates hardness, dimensional accuracy, and wear resistance of the SLM fabricated tool inserts through the injection of 150,000 parts.

## 2. Materials and Methods

### 2.1. Overall Framework

The framework methodology employed in this study is structured to outline the major steps this research work follows: firstly, the four Stainless Steel 316 L tool inserts required for investigating this study were fabricated simultaneously using SLM technology. After the tool inserts were built and removed from the build chamber, microstructure analysis was conducted to explore particle formation of the laser melted specimens, layer structure, and chemical composition. Three types of tests were managed: optical microscopic inspection using a Carl Zeiss Axiovert 200 microscope, SEM inspection using an ultra-high-resolution Leo Supra 55, and EDS analysis. After the microstructure inspection and analysis was successfully investigated, more tests on the fabricated tool inserts were required. The purpose of these tests was to examine micro-hardness and dimensional accuracy of the fabricated tool inserts prior to use in the injection moulding process. Microhardness was achieved using a Leco Vickers micro-hardness tester with a square-based diamond pyramid indenter and 10 Kg load subjected to each half of the tool inserts with a dwell time of 15 s. Each tool insert were categorised into batches and a sample of products was inspected for dimensional accuracy and functionality. At stage two of injection moulding, the first tool insert was excluded, and the remaining three inserts were mounted on the same bolster to continue production until 20,000 injections were completed. The same tests that were performed at previous stages were conducted after the tool inserts were dismounted. Sampling and inspection of dimensional accuracy and functionality of the produced parts were implemented at this stage. The same procedure was followed for the remaining two tool inserts by removing the second tool insert and examining the third and fourth tool inserts and their respective products. After the fourth tool insert successfully achieves 40,000 injections, injection moulding was continued to attempt to reach the goal of producing 150,000 dimensionally accurate, and functionally approved products.

### 2.2. Tool Insert Fabrication

**Figure 1:**
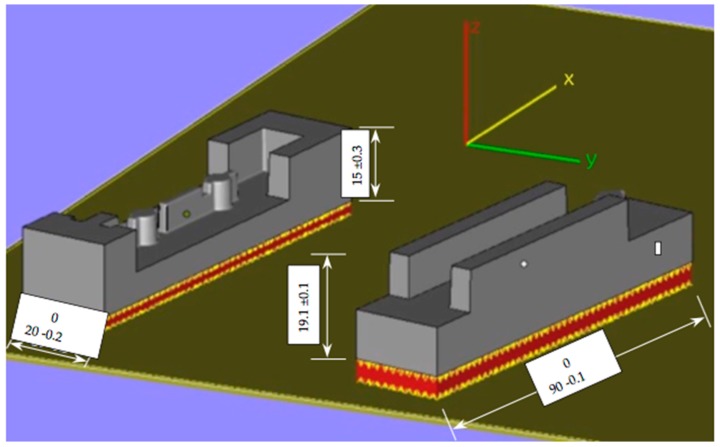
Part orientation, layer structure, and main dimensions (mm) during sintering process.

**Figure 2:**
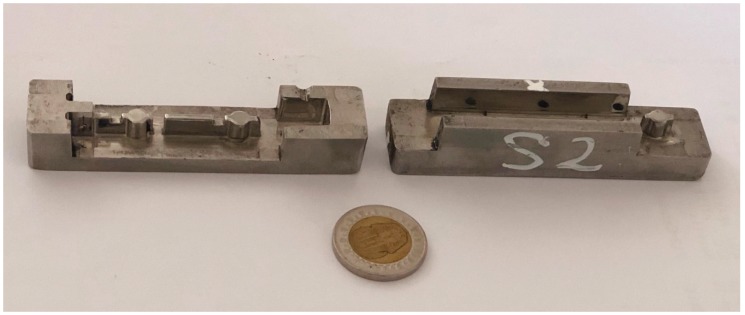
One set of SLM fabricated Core and Cavity tool inserts.

SLM was used for fabrication of four sets of tool insert specimens directly from 3D CAD data models at an automotive spare-parts manufacturing company. The build was conducted on a Realizer SLM 250 with a laser power of 200W and build orientation as shown in Figure 1. The maximum part dimensions were 90 mm × 20 mm × 15 mm. The final fabricated tool insert core and cavity are shown in Figure 2. Parts were scaled in the CAD model to compensate for allowances caused by shrinkage during cooling of the injected products. Stainless Steel 316L powder was the material in use for the builds, with particle size nominally in the range of 45–150 μm and a layer thickness of 50 μm.

During the SLM fabrication process, the hatch distance defined as the spacing between two consecutive laser beams, identified that the hatch X and Y distance was set at 0.1 mm respectively. Initially, sand blasting was used to remove the excess powder after the fabrication process to ensure accurate surface mating of the Cores with the Cavities of each set of inserts. However, experimental procedures were carried out to investigate microstructure and chemical composition. Further investigations were performed prior to and after injection moulding in the context of microhardness analysis and geometrical accuracy.

## 3. Tool Experimentation, Results and Discussion

### 3.1. Microstructure

Four sets of tool inserts were prepared for inspection by optical microscopy. The parts were wet smoothed using a linishing belt grinder with 180 grit abrasive sandpaper for approximately 5 min for each part. The samples were further polished successively with 220 and 1000 grit abrasive sandpaper to acquire the necessary surface finish. To maintain a glossy look, a polishing paste (Microid Diamond Compound, LECO Corp., St. Joseph, MI, USA) was applied to the surfaces and rubbed with a smooth cloth.

**Figure 3:**
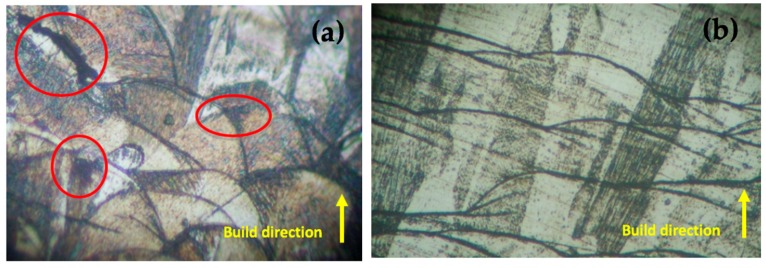
200x (**a**) and 500x (**b**) magnification of inspected specimen on Carl Zeiss Axiovert 200 with evidence for presence of carbide inclusions.

To reveal the microstructure, the polished samples were immersed in a chemical acidic solution for 20 min; the solution contained 96% pure white Alcohol, 2% Nitric Acid (with a concentration of 69%) and 2% Hydrochloric Acid. After removal from the solution, the specimens were cleaned in distilled water. Inspection of the specimens suggested the presence of carbides and porosity along the surface of the layers. Images were captured and magnified to 200x and 500x respectively. Image capturing was repeated three times for each of the five regions of interest chosen on the same specimen to confirm the evidence that higher contents of carbides are detected. The elemental chemical composition of the fabricated specimens was determined using a Spectral Analyser as shown in Table 1. Captured images of the magnified surface are shown in Figure 3.

**Figure 4:**
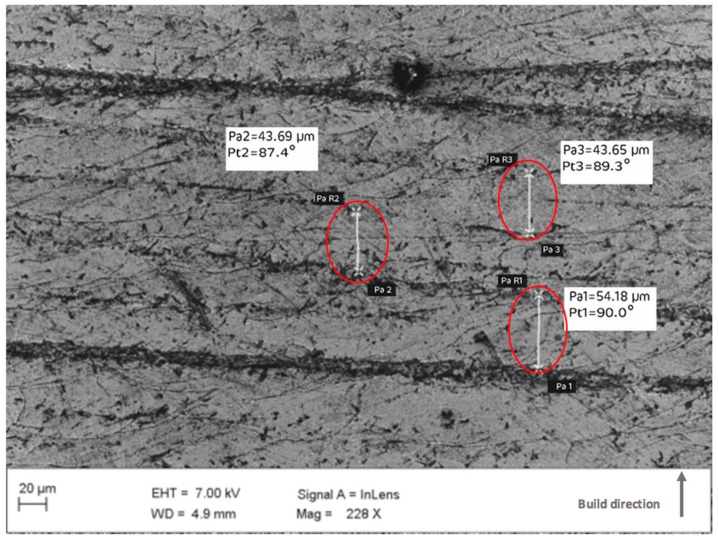
SEM micrographs of SLM tool insert surface with recorded layer thickness at three different points.

SEM with a linked EDS system was employed to observe particle formation, layer structure, chemical composition, surface morphology, and microstructure of the laser melted specimens. As highlighted in Figure 3, the presence of intermetallic carbides is concentrated in some regions more than others along the layer surface of the sintered specimens. Three measurements of the layer thickness were recorded for a particular region of the layer as shown in Figure 4. The average recorded layer thickness is 47.17 μm at 228x magnification. The procedure was repeated three times for each region, with five separate regions considered.

**Figure 5:**
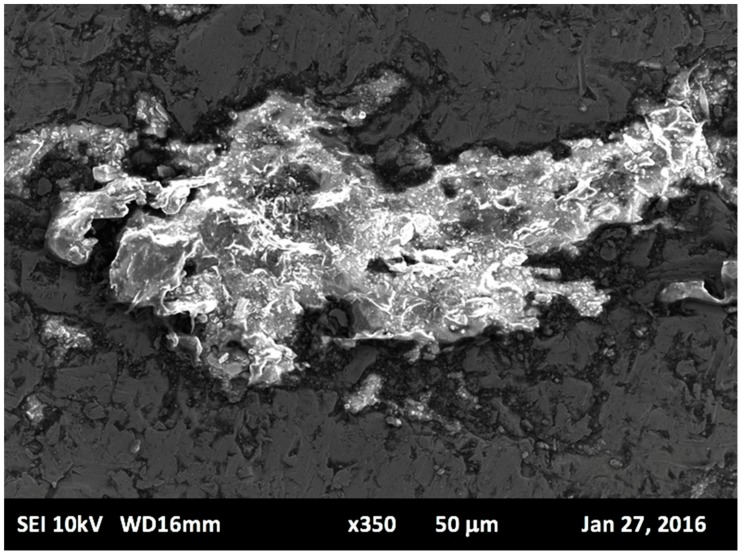
Carbide segregation due to layer boundary.

Images captured from the SEM provide significant evidence that formation of intermetallic carbides is present along the layer surface of the sintered specimens. Carbide formation is concentrated in some regions more than others, specifically along the boundary of each individual layer. During the laser melting process, the presence of high concentrated weights of chromium, nickel, and molybdenum in Stainless Steel 316L allows carbides to form resulting in comparably superior mechanical properties. Particularly, higher microhardness, enhanced tensile strength, fatigue life, and good corrosion resistance, as compared to commercial Stainless Steel 316 L [44]. The prospect of knowing the elemental type of intermetallic particle that is formed involves extensive analysis. An EDS system was employed to detect the type and size of intermetallic particles that may cause carbide formation. Quantitative analysis of the alloying elements of Stainless Steel 316L was conducted. Figure 5 shows a micrograph of the presence of intermetallic particles along the layer boundary. Images captured are magnified to 150x. The image capture process is repeated three times for the specified region, with five separate regions considered. At different magnifications using SEM, several significant features were discernable. At low magnification, layer melt pool alignment was observed, whilst at higher magnification, intersection between two-layer melt pools revealed a cellular structure and carbide formation.

**Figure 6:**
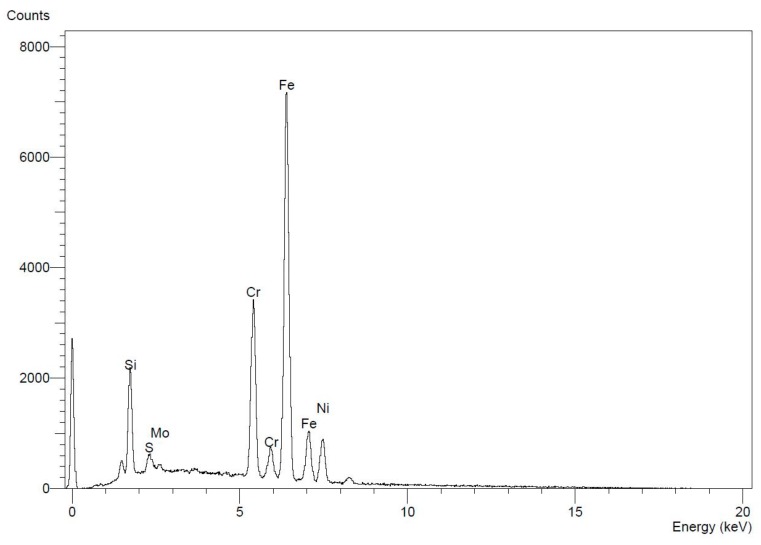
Elemental analysis using EDS for intermetallic particles segregated towards cell boundary, and demonstrating the enrichment of Cr, Ni, and Mo at the boundaries.

Figure 6 provides a summary of the results from the EDS analysis. The data indicates the type of intermetallic particle with the highest concentration level is Chromium accounting for 4000 intensity counts. Nickel accounts for 1000 counts, and Molybdenum has the lowest concentration level of 500 counts.

The results obtained from the optical microscopy test revealed that after inspection of the specimens, the presence of carbides and porosity along the surface of the layers is noticeable. The data obtained from the spectral analysis test matches with the standard acceptable range for Stainless Steel 316L [43]. Moreover, EDS analysis results confirmed the presence of highly concentrated areas of chromium, nickel, and molybdenum in Stainless Steel 316L allowing carbides to form resulting in reinforcements of some mechanical properties. Summarized in Figure 6, the data indicates the type of intermetallic particle with the highest concentration level. The highest concentration level accounted for was Chromium followed by Nickel, and the lowest concentration level was Molybdenum. Carbon was not counted nor classified in the EDS measurement, because it should only account for less than 2 wt.% of the chemical composition of the material, which is in good agreement of the alloy balance [45]. However, Silica is accounted for with a high concentration level due to the presence of an impurity within the formed carbide particle.

It is well known that during the SLM fabrication process, melt pools are created. Therefore, it was observed using SEM that the melt pools are aligned in an interlacing arrangement as a result of laser scanning patterns and rapid solidification, therefore a distortion to grain structure and boundaries causes considerable difference to microstructure scales for sintered stainless steel 316L [46].

### 3.2. Hardness Test

A micro-hardness test was employed to determine the Vickers hardness for the SLM fabricated specimens. The micro-hardness test was performed at two different stages of the research to determine the potential variation to hardness as a consequence of the thousands of impressions from continual injection moulding cycles. The first stage of micro-hardness tests was performed individually for the four sets of tool inserts after SLM fabrication and before the tool inserts were mounted for injection moulding. The second stage for testing micro-hardness of the four sets of tool inserts was conducted after the injection moulding process was completed.

**Figure 7:**
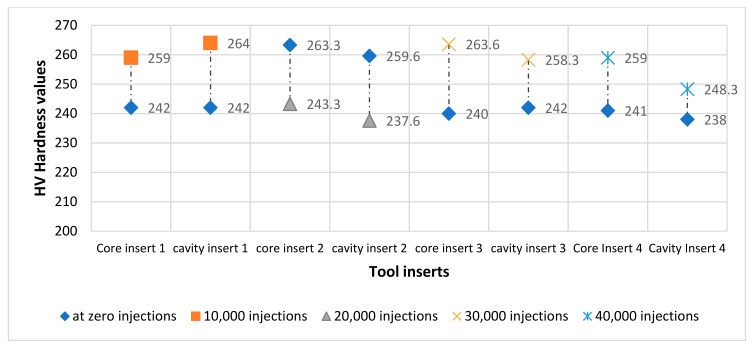
Changes in micro-hardness of the SLM specimens depicts variation prior to and after injection moulding using each tool insert.

For each specimen, two measurement points were recorded to monitor variation in hardness values before and after injection moulding. The values recorded are the resultant average of three readings from the same region. Figure 7 illustrates the changes observed in the hardness values according to the stage in which the test was performed. The Core and Cavity halves of tool insert set 1 have comparable values of 242 HV when tested prior to injection moulding. After 10,000 injections, tool insert set 1 was dismounted and further micro-hardness tests were undertaken. For the Core half, the hardness value had increased to 259 HV and the Cavity half increased to 264 HV. For the second set of tool inserts the same test procedure was conducted, the Core and Cavity had hardness readings of 243.3 HV and 237.6 HV respectively. After 20,000 injections, the second tool insert was dismounted, and hardness tests were performed. The Core hardness value increased to 263.3 HV, while the Cavity increased to 259.6 HV. The Core and Cavity hardness readings before commencing injections for the third tool insert were 237.6 HV and 240 HV respectively. At 30,000 injections, the third tool set is dismounted, the Core hardness reading increased to 263.6 HV and for the Cavity the hardness value increased to 258.3 HV. The fourth tool insert set recorded hardness values of 241 HV and 238 HV for the Core and Cavity respectively before injections. After 40,000 injections, the hardness value for the Core insert increased to 238.3 HV and 248.3 HV for the Cavity.

The preset value on the machine for layer thickness is set at 50 μm, but it is known that variation in layer thickness can result due to heat dispersions along the built layer. Since increasing layer thickness increases the porosity, hardness eventually decreases with this increase in layer thickness [47]. Moreover, the presence of gas pores depending on their shape and size are also expected to cause defects on the surface in the form of surface porosity. Therefore, the specimens were examined for porosity inclusions.

It is noted that there is a minor increase to the hardness value from the initial material before injection moulding is commenced. This increase in hardness could be explained due to changes of the temperature to which the tool is exposed during processing which has a strong influence on the phase composition, the microstructure and the mechanical performance of 316L stainless steel [48].

### 3.3. Dimensional Measurements

**Figure 8:**
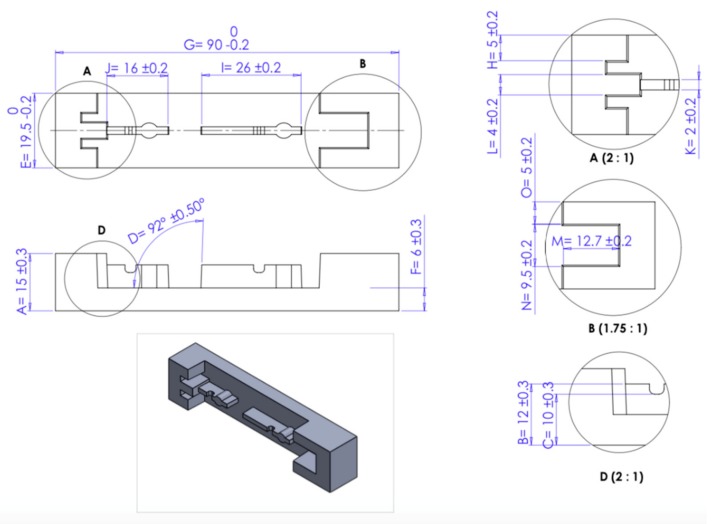
SLM Core measurements (with tolerances indicated).

**Figure 9:**
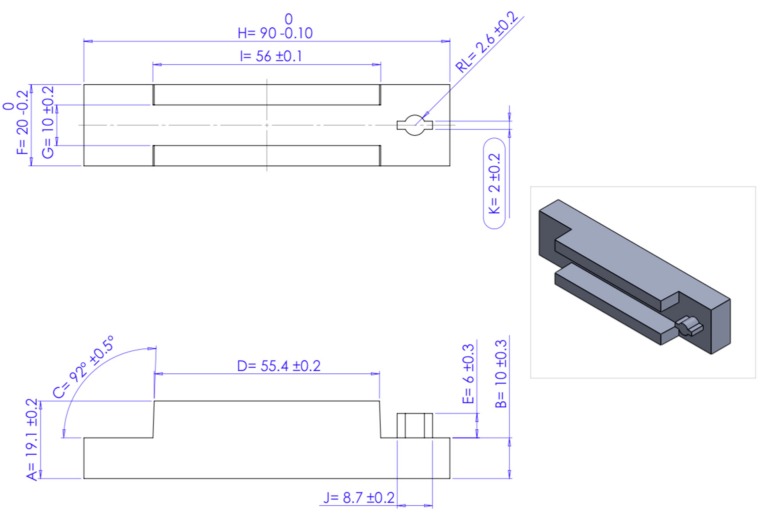
SLM Cavity measurements (with tolerances indicated).

Further analysis is necessary to determine deviation in measurements from the nominal values after the tool inserts are fabricated, to detect the existence of wear. Polypropylene was the material used for the injected products, so a 1.5 % shrinkage allowance for injection moulding is compensated for during the design stage. Specific dimensional measurements were accounted for in each Core and Cavity of the four SLM tool insert sets. A Zeiss Abbe Horizontal Metroscope and a Zeiss Universal Measuring Machine were used for measuring the dimensional accuracy of the specimens. The tolerances were set according to the automotive spare-parts manufacturing company’s standards for tool manufacturing. The dimensions for each Core and Cavity are illustrated in Figure 8 and Figure 9 for the four sets of tool inserts. Dimensional accuracy of all the tool inserts was examined for each of the 15 Core dimensions and the 12 Cavity dimensions specified. Each is the resultant average of three measurements for the same dimension. Four internal and external dimensions were investigated for each tool insert and the dimensions noted are a representation of the rest of the dimensions and their outcomes. The four dimensions selected for this study are dimensions I and N shown in Figure 8 (Core), and dimensions E and G shown in Figure 9 (Cavity). Table 2: lists the dimensions used for measurement assessment of the tool inserts.

Measurements were recorded for the four sets of tool inserts after the SLM process and before injection moulding was initiated. Measurements taken before injection for Core halves 1, 2, 3 and 4 are within the range of permissible design tolerance. After 10,000 injections are completed for Tool 1, the Core and Cavity inserts are dismounted, and further dimensional examination is required. After 20,000 injections on tool 2, the same procedure was repeated, and the Core and Cavity inserts were dismounted for further dimensional examination. For tool inserts 3 and 4, the same procedure was repeated by dismounting the third set of tool inserts after 30,000 injections and the fourth tool inserts after 40,000 injections for further dimensional examination.

**Figure 10:**
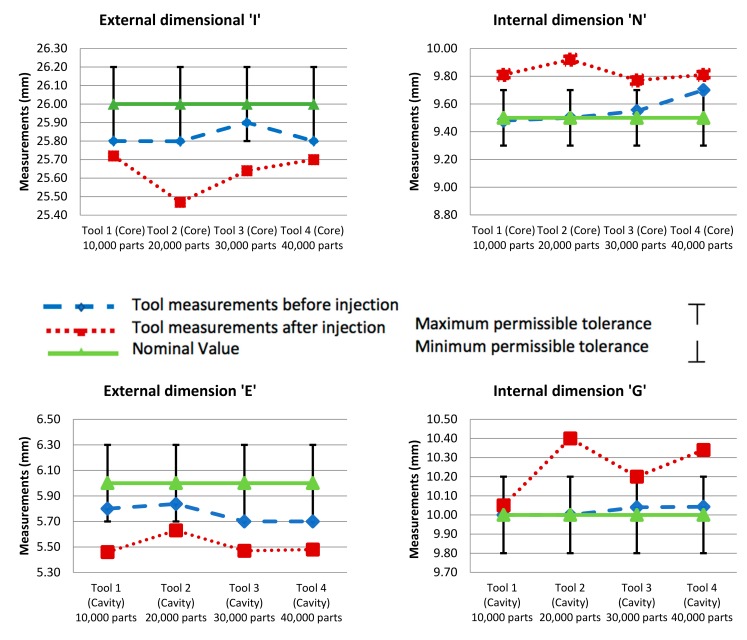
Dimensional measurements of Core and Cavity of the tool inserts with upper and lower tolerances.

Dimension I of the Core inserts is an external dimension and has a nominal value of 26 mm and a design tolerance of ± 0.2 mm. Dimension N of the Core inserts is an internal dimension with a nominal value of 6 mm, with a permissible design tolerance of ± 0.2 mm. For the tool Cavities, dimension E is external with a nominal value of 6 mm and ± 0.3 mm design tolerance. Dimension G is an internal dimension of the tool Cavities, the nominal value is set at 10 mm with a ± 0.2 mm design tolerance. Table 3 illustrates the recorded measurements of dimensions I, N, E, and G before and after the injection moulding process and the deviation from the upper and lower permissible tolerances of each dimension. Figure 10 demonstrates dimensional measurements of the tool inserts with upper and lower tolerances.

It is noted that changes in dimensional accuracy are interpreted as progressive wear due to the many thousands of components produced through the injection moulding process. For external dimension I all four Cores were subjected to wear in addition to deviation from the lower maximum permissible tolerance. As for internal dimension N, all four cores deviated from the upper permissible tolerance. For the tool Cavities, recorded values of the measurements of external dimension E taken after the injection moulding indicates that the Cavities have experienced wear deviating from the lower permissible tolerance. As for internal dimension G, measurements documented for two of the four Cavities, cavity of tool 1 and 3 show that the values are within the acceptable tolerance range. However, cavities of tool 2 and 4 are beyond the upper permissible tolerance range. After analysing the recorded data for measurements taken before and after injection moulding, it was noted that wear does increase as the number of injections increase, but not necessarily in a consistent ratio to the number of injections. However, changes in dimensional accuracy are sufficient to confirm that the tools are susceptible to wear due to the progressive and continued loads exerted on the tools by the injection moulding process. Additionally, it is noted that for external dimensions deviation from the accepted tolerance tends to surpass the lower permissible range. Contrary to internal dimensions, deviation tends to surpass the upper permissible tolerance.

## 4. Product Evaluation of Injection Moulding

Evaluating the SLM-fabricated tool inserts was implemented through injection moulding. The parts produced were investigated to analyse dimensional accuracy, surface quality, and product functionality.

### 4.1. Injection Moulding

The injection moulding was conducted using a Nurnak MMRJ 130-225 moulding machine with clamping force of 100 ton. Polypropylene was chosen as the material for injection moulding with a stock feed rate of 25 g/stroke, injection pressure 75 bar and the temperature maintained constant at 220 °C. During the injection moulding process, the tool inserts temperature was constantly monitored using an infrared heat detector and maintained at 20 °C to avoid overheating. The melt temperature was controlled to ensure consistency and uniformity of the process parameters.

### 4.2. Dimensional Accuracy of Injection Parts

**Figure 11:**
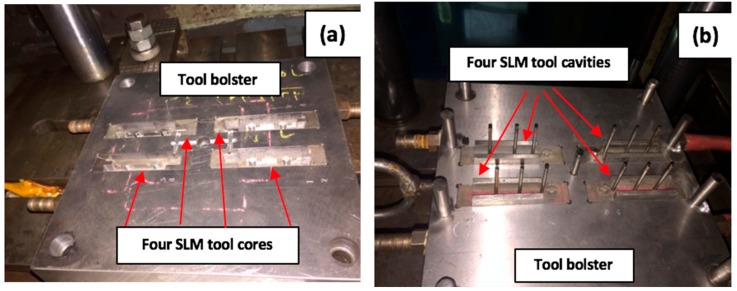
Four sets of tool inserts mounted on the bolster (**a**) Four SLM core inserts (**b**) Four SLM cavity inserts.

The four sets of tool inserts were installed within the same bolster using the same working conditions to ensure parametric consistency. The steel mould base plates were machined with rectangular pockets to fit the tool inserts within. Figure 11 shows the position of the inserts after they were mounted onto the bolster. The average cycle time was calculated to be approximately 34 s. 19 g was the total weight of the product tree with four components attached, with the net weight of each component produced being 4 g.

The injected products were grouped into smaller batches for each run. When injection is initiated, polypropylene is rapidly forced into the tool Cavities and as a result, a sudden pressure increase is exerted on the tool inserts. This pressure increase is the highest pressure reached during the injection process. Therefore, after thousands of successive injections, the applied force on the Core features may cause fractures, cracking or wear on the tool inserts that will eventually change the dimensional accuracy of the parts produced. A number of the components were selected by way of sampling, to analyse possible variations in dimensional measurements as the injection moulding process progressed.

It is certain that product measurements are required to prove accuracy of the tool inserts. However, measuring the entirety of the product output (i.e., tens of thousands of injected components) was not realistic, therefore a sample size was required to represent the targeted population. The sample sizes are determined based on a sampling Equation [49] as follows:

n=Zα2E2

where, n = sample size, Z = standard normal score from the normal curve table [49] based on the degree of confidence interval, E = maximum permissible error depending on population, Confidence interval = 90% and α = 0.1.

**Figure 12:**
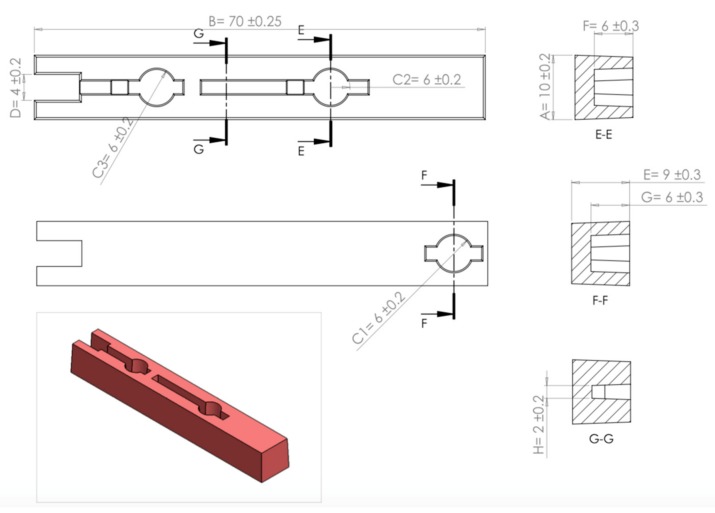
Injected part illustration with dimensional measurements and tolerances.

The sampling equation is used to determine the optimum sample size for each of the four runs. The maximum permissible error varies from each run depending on the increase in product population. Values were set with consideration regarding the number of samples to be selected with a tradeoff between the time taken to measure each sample and the cost of measuring them. For runs 1 to 4, the maximum permissible error was set at 0.2, 0.15, 0.1 and 0.1 respectively. Production runs are categorised into four runs depicting batching of 10,000, 20,000, 30,000, and 40,000 parts per run. For each run, a number of samples were randomly selected for functional inspection and to ascertain dimensional accuracy. Therefore, the number of samples to be selected as calculated by the sample equation for each run were as follows: 42, 80, 120, and 166 samples respectively. Each run is divided into smaller batches, for each batch, two samples are randomly selected for measurement and the average value is taken for those two values. Therefore, the average value calculated is recorded for each batch. The recorded values are 21, 40, 60, and 83 respectively for each run. Figure 12 is an illustration of the part dimensions to be measured and their nominal values. Two dimensions D and H of the parts produced, are selected for discussion in this paper, the selected dimensions and their outcomes being representations of the remaining unstated dimensions.

**Figure 13:**
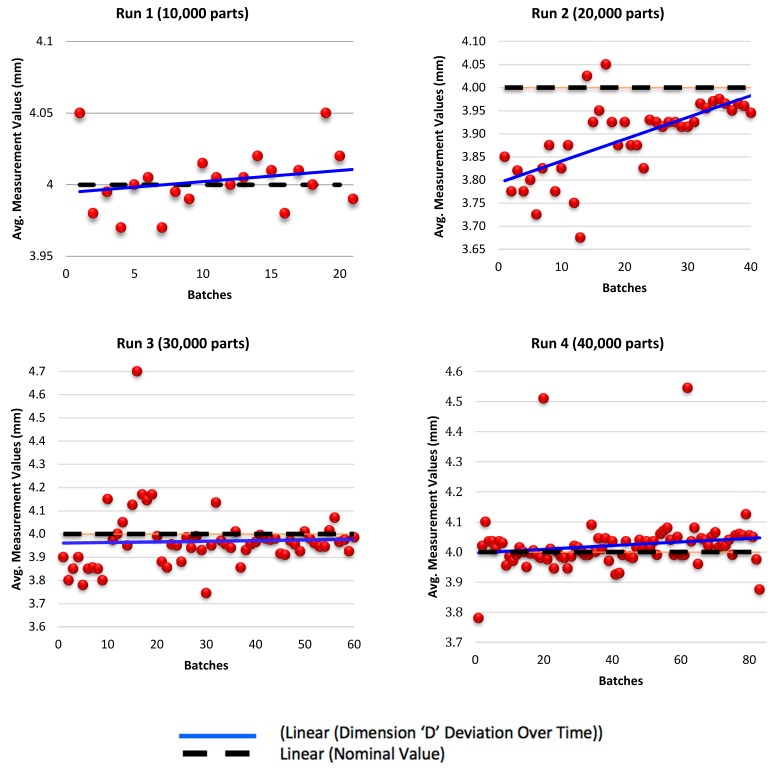
Sample measurements for dimension ‘D’ deviation over time for run 1, run 2, run 3, and run 4.

Figure 13 demonstrates dimensional deviation for dimension D (internal dimension) over time. A ±0.2 mm design tolerance is set to ensure acceptability of the part as an end product. Most of the recorded values of the four batches were defined to be within the acceptable tolerance range of the measurements. However, for runs 2, 3 and 4, a few outlier batch values were spotted dispersing outside the limit zone, and these values were considered negligible in comparison to the values of the rest of the batches.

**Figure 14:**
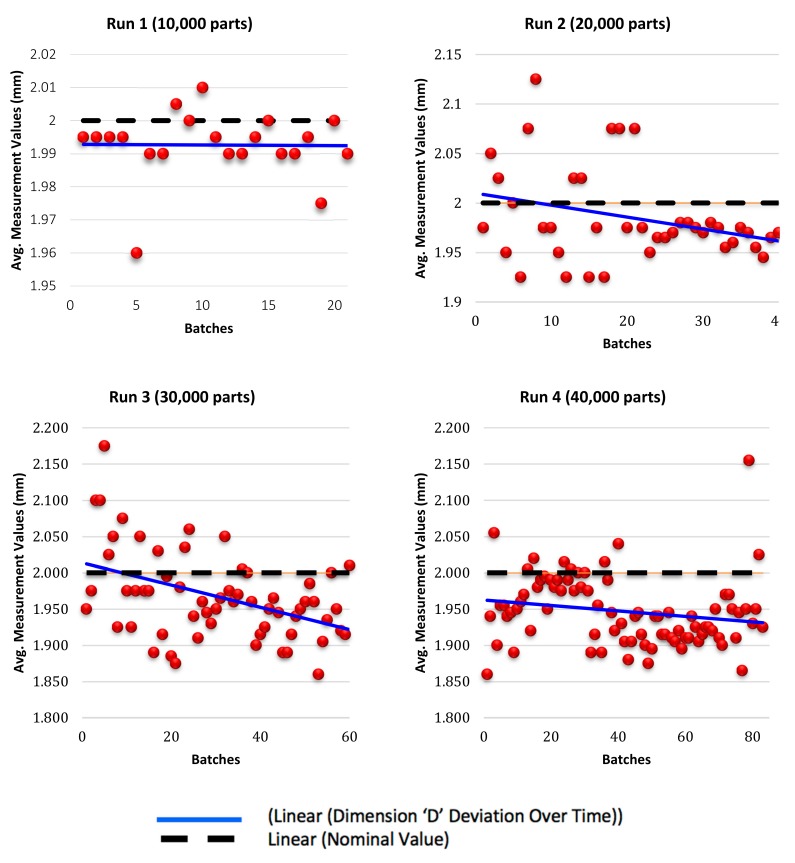
Sample measurements for dimension ‘H’ deviation over time for run 1, run 2, run3, and run 4.

Measurement values for Dimension H (external dimension) are illustrated in Figure 14 for runs 1, 2, 3 and 4 respectively. A ±0.2 mm design tolerance is set to ensure acceptability of the part as an end product. The recorded measurements were within the acceptable tolerance range.

For dimension D, most of the recorded values of the four batches were defined to be within the acceptable tolerance range of the measurements. It was noted that as injection moulding progresses, recorded values of the batches demonstrate a direct linear regression trending towards the nominal value. In conclusion, a positive linear regression of the measurement values of internal dimension D depicts the development of wear on the specified tools as a function of the number of progressive injections. It was noted that measured values at the beginning of injection moulding were widely scattered and gradually drifted towards the acceptable range within the limits of the nominal values.

For dimension H, the recorded measurements were within the acceptable tolerance range. Moreover, data formation along the trend line represents a negative linear regression that emphasises the direct relation between the progression of wear and the number of samples. Kanagarajah et al. [50] discussed that elemental segregation of intermetallic particles has significant impact on the corrosion characteristics as well as wear resistance along the built layers, yet strength is adversely affected causing the material to be brittle which may have an unfavourable effect on tool insert longevity.

### 4.3. Injection Moulding of 150,000 Parts

Previous research was reviewed by Nagahanumaiah [51] and it was clearly stated that there has been no published work on the quality and effect of injection moulding on Direct Metal Laser Sintering (DMLS) fabricated tools – their work was completed through the production of 5000 parts. The work of Dolinšek [52] indicated the recommendations made by EOS Manufacturing Solutions that metallic moulds are capable of withstanding 100,000 injections but with no practical proof to indicate tool life performance and wear resistance. Therefore, this study was directed to successfully accomplish the production of 150,000 injections from the SLM fabricated tool insert, ensuring that no damage will occur to the tool inserts after successive tens of thousands of injections.

**Figure 15:**
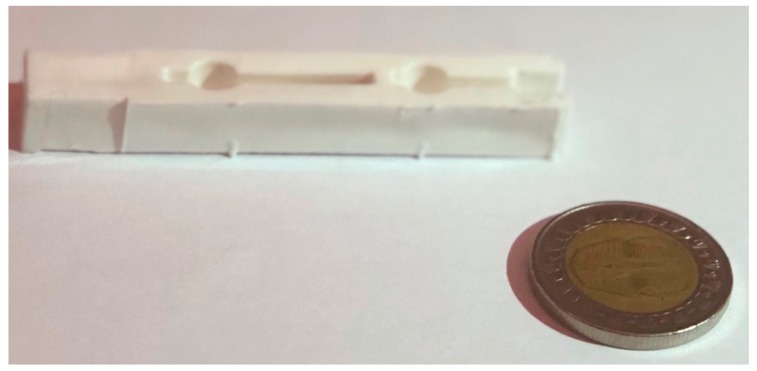
Sample batch production of 1000 components of injected parts.

40,000 injections was the initial limit reached for the fourth tool set and no signs of fracture, cracks, or wear were noticeable. Therefore, a new goal was set to further guarantee that the fourth tool insert could withstand more injections runs. The goal was to reach 150,000 injections in total with no apparent failure to either the tool set or the components produced. As 40,000 components were already produced from the fourth tool set, a further 110,000 additional injections were to be produced for the purpose of completing 150,000 components in total. Each run was set to produce 10,000 components, each batch being divided into smaller volumes of 1000 components and labelled consecutively from 1-1000, 1001-2000 and so forth. Figure 15 displays the produced part.

**Figure 16:**
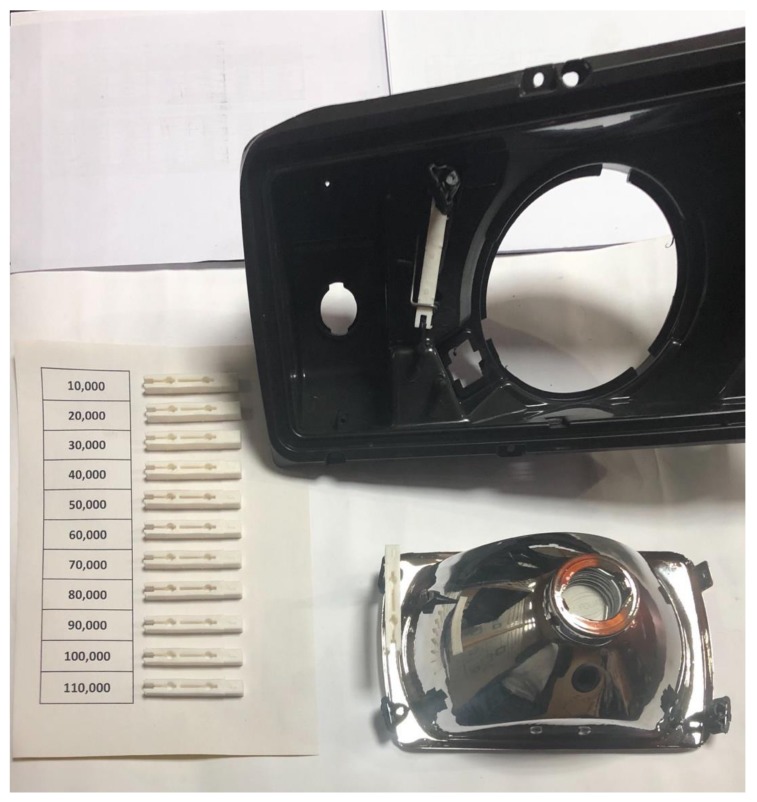
Sample demonstration of visual inspection and component fitting to headlight housing.

The same sampling equation is used to determine the optimum sample size for the production runs. The maximum permissible error was set at 0.1. Therefore, the number of samples to be selected for each run was 170 samples. For each batch of 1000 components, 17 samples were randomly selected for visual inspection and fitting. Figure 16 displays one sample from each of the eleven runs after injection of 10,000 from each run. From each run, 170 samples were selected for inspection, the parts inspected and compared together to identify if there were any significant defects. After the inspection process, the parts were fitted to the headlamp housing to ensure product functionality. Functional success is perceived through successful assembly of the part produced from the injection moulding process to the headlamp, this is achieved based on an industrial quality control procedure to ensure the functionality of the end-use product through ease of assembly and accurate fixation of the part. Shown in Figure 16 is a sample illustration of the fitting process. As a result, the parts were deemed acceptable in terms of visual inspection and product functionality. Moreover, the samples appear to be in an acceptable shape showing no signs of flash or over-moulding, cracks or surface imperfections. Therefore, the tool insert proved to remain faultless, and it is expected could continue production of multiple hundreds of thousands before failure might occur.

Previous work reviewed by Nagahanumaiah and Dolinšek [51,52] expressed uncertainties related to SLM capabilities in fabricating injection moulding tools that can be used for high-volume production of thousands of parts, referring to limitations of the SLM technology in producing functional products with high quality as opposed to conventional injection moulding. However, after the tool inserts proved to be successful in producing tens of thousands of functional products without failure, more production runs were initiated to guarantee longevity of the tool inserts. The fourth tool insert that produced 40,000 products continued production until 150,000 parts were produced. The number of samples selected for inspection for each of the eleven runs was 170 samples. Parts were visually inspected and functionally approved through fitting the parts in the headlight’s housing to ensure product validity. The parts proved to be functional and visually acceptable showing no signs of defects. Therefore, the tool insert proved to be in a faultless form, and it is expected could continue production of multiple hundreds of thousands more parts before failure occurs.

## 5. Conclusions

Experimental work conducted on the four stainless steel 316L tool inserts fabricated using SLM lead to the following conclusions:

- Microstructure and EDS analysis confirmed the inclusion of a high content of carbides along the edge of each individual layer. The elements with the highest concentration were Chromium, Nickel, and Molybdenum respectively. Therefore, the existence of carbides caused by the laser melting process resulted in reinforcing microhardness and projected a positive outcome for durability due to elemental segregation.
- For the first reported time, SLM fabricated tool inserts proved to be successful in performance with regard to injection moulding of 150,000 parts. The four tool insert sets were run for 10,000, 20,000, 30,000, and 40,000 injections respectively. Finally, after the fourth tool insert successfully completed 40,000 injections, further production runs were continued to achieve 150,000 injections. It was proven that the fourth set of tool inserts was able to withstand 150,000 injections without any significant signs of failure.
- Wear is acknowledged as a result of the progression of the injection moulding process. However, steadiness in the wear rate was noted amid large production runs. Alterations to dimensional accuracy verifies that the tool inserts are liable to wear due to successive loads by the injection moulding process.
- It is concluded from the work done in this research that additive manufacturing SLM technology proved to be a reliable technique for fabricating Stainless steel 316 L injection moulding tool inserts for the aftermarket automotive industry.

## Figures and Tables

**Table 1:** Stainless Steel 316L elemental weights (Wt%).

Wt %									
Sample	C	Si	Mn	P	S	Cr	Mo	Ni	Fe
SS 316 L Standard [43]	0.035	0.75	2	0.045	0.03	16-18	2-3	10-14	Balance
SLM	0.024	0.41	1.52	0.023	0.021	16.057	2.38	10.397	Balance

**Table 2:** Dimensions indicators used for measurement assessment of tool inserts.

Dimension	Location	Type
I	Core	External
N	Core	Internal
E	Cavity	External
G	Cavity	Internal

**Table 3:** Dimensional measurements before and after injection process and deviation from permissible tolerances (mm).

	Dimension I			Dimension N		
Tool Number	Measurements before Injection (mm)	Measurements after Injection (mm)	Deviation from Permissible Tolerance (mm)	Measurements before Injection (mm)	Measurements after Injection (mm)	Deviation from Permissible Tolerance (mm)
Tool 1 10,000 parts	25.8	25.72	−0.08	9.48	9.81	0.11
Tool 2 20,000 parts	25.8	25.47	−0.33	9.50	9.92	0.22
Tool 3 30,000 parts	25.9	25.64	−0.16	9.55	9.77	0.07
Tool 4 40,000 parts	25.8	25.70	−0.10	9.70	9.81	0.11
	**Dimension E**			**Dimension G**		
**Tool Number**	**Measurements before Injection (mm)**	**Measurements after Injection (mm)**	**Deviation from Permissible Tolerance (mm)**	**Measurements before Injection (mm)**	**Measurements after Injection (mm)**	**Deviation from Permissible Tolerance (mm)**
Tool 1 10,000 Parts	5.80	5.46	−0.24	10.0	10.05	0.05
Tool 2 20,000 parts	5.84	5.63	−0.07	10.0	10.4	0.4
Tool 3 30,000 Parts	5.67	5.47	−0.23	10.04	10.2	0.16
Tool 4 40,000 Parts	5.67	5.48	−0.22	10.04	10.34	0.3
